# MiR-153 Regulates Amelogenesis by Targeting Endocytotic and Endosomal/lysosomal Pathways–Novel Insight into the Origins of Enamel Pathologies

**DOI:** 10.1038/srep44118

**Published:** 2017-03-13

**Authors:** Kaifeng Yin, Wenting Lin, Jing Guo, Toshihiro Sugiyama, Malcolm L. Snead, Joseph G. Hacia, Michael L. Paine

**Affiliations:** 1 Center for Craniofacial Molecular Biology, Herman Ostrow School of Dentistry, University of Southern California, Los Angeles, CA, USA; 2 Department of Orthodontics, Herman Ostrow School of Dentistry, University of Southern California, Los Angeles, CA, USA; 3 Department of Endodontics, Herman Ostrow School of Dentistry, University of Southern California, Los Angeles, CA, USA; 4 Department of Biochemistry, Akita University of Graduate School of Medicine, Hondo, Akita, Japan; 5 Department of Biochemistry and Molecular Biology, Institute for Genetic Medicine, Keck School of Medicine, University of Southern California, Los Angeles, CA, USA

## Abstract

Amelogenesis imperfecta (AI) is group of inherited disorders resulting in enamel pathologies. The involvement of epigenetic regulation in the pathogenesis of AI is yet to be clarified due to a lack of knowledge about amelogenesis. Our previous genome-wide microRNA and mRNA transcriptome analyses suggest a key role for miR-153 in endosome/lysosome-related pathways during amelogenesis. Here we show that miR-153 is significantly downregulated in maturation ameloblasts compared with secretory ameloblasts. Within ameloblast-like cells, upregulation of miR-153 results in the downregulation of its predicted targets including Cltc, Lamp1, Clcn4 and Slc4a4, and a number of miRNAs implicated in endocytotic pathways. Luciferase reporter assays confirmed the predicted interactions between miR-153 and the 3′-UTRs of Cltc, Lamp1 (in a prior study), Clcn4 and Slc4a4. In an enamel protein intake assay, enamel cells transfected with miR-153 show a decreased ability to endocytose enamel proteins. Finally, microinjection of miR-153 in the region of mouse first mandibular molar at postnatal day 8 (PN8) induced AI-like pathologies when the enamel development reached maturity (PN12). In conclusion, miR-153 regulates maturation-stage amelogenesis by targeting key genes involved in the endocytotic and endosomal/lysosomal pathways, and disruption of miR-153 expression is a potential candidate etiologic factor contributing to the occurrence of AI.

Amelogenesis, the process of enamel formation, involves two major functional stages–secretory and maturation^1^. As enamel organs transition from secretory to maturation stage, the innermost layer of cells–ameloblasts–undergo both morphological and functional changes. Ameloblasts in secretory stage mainly function to synthesize and secrete a series of enamel matrix proteins (EMPs) into the future enamel space^2^. During enamel maturation, EMPs are fragmented extracellularly in the presence of ameloblast-derived proteinases^3^,^4^,^5^,^6^. The EMP debris is retrieved by ameloblasts for further degradation within the cellular endosomal/lysosomal apparatus^7^,^8^,^9^, and the concomitant ion flux through the ameloblasts gives rise to mature tooth enamel with highly organized crystal structure. Thus, removal of EMPs mediated by ameloblast-hosted endocytotic and endosomal/lysosomal pathways is one of the key processes in maturation-stage enamel formation^7^,^10^,^11^. Although earlier studies focused on passive diffusion or non-specific internalization in interpreting the cellular uptake of EMPs from enamel space^12^,^13^,^14^,^15^,^16^, more evidence from recent studies lends support to a mode of receptor-dependent endocytosis with coated vesicles^7^,^8^,^9^,^10^,^11^.

The involvement of miRNAs in tooth development was first proposed in 2008^17^. Two functional studies, in which epithelial Dicer-1 was deleted at earlier stages of tooth development, elicited tooth phenotypes with varying severities^18^,^19^. *In vitro* studies confirmed the functional roles of miR-34a, miR-143 and miR-145 in the regulation of terminal dental cell differentiation^20^,^21^. Furthermore, the expression of miRNAs showed a dynamic property during different stages of tooth formation^11^,^19^,^22^. In our previous study, we performed genome-wide miRNA and mRNA transcriptome analysis using RNA samples isolated from rat secretory- and maturation-stage enamel organs^11^. We identified a group of miRNAs expressed in a stage-specific manner, which are significantly enriched in the gene functional categories equivalent to those key processes during amelogenesis. Most notably, miR-153 is predicted to be one of the miRNA regulators targeting endocytotic and endosomal/lysosomal pathways, and experimental evidence from luciferase report assays validated the predicted interaction between miR-153 and the 3′-UTR of Lamp1^11^.

Here, we further extended our knowledge about the role of miR-153 in amelogenesis and the pathogenesis of Amelogenesis Imperfecta (AI) by collecting additional evidence from both *in vitro* and *in vivo* functional studies. We identified key genes in the endocytotic and endosomal/lysosomal pathways that were regulated by miR-153 expression in our assay systems. In addition, we provide evidence of the importance of miR-153 expression levels in regulating amelogenesis and the specific molecular mechanisms that miR-153 plays in this process. Our findings would help better understand the potential etiologic factors contributing to the occurrence of AI and possible preventative or therapeutic approaches to disease.

## Results

### The expression of miR-153 is significantly downregulated during maturation-stage amelogenesis

**Figure 1:**
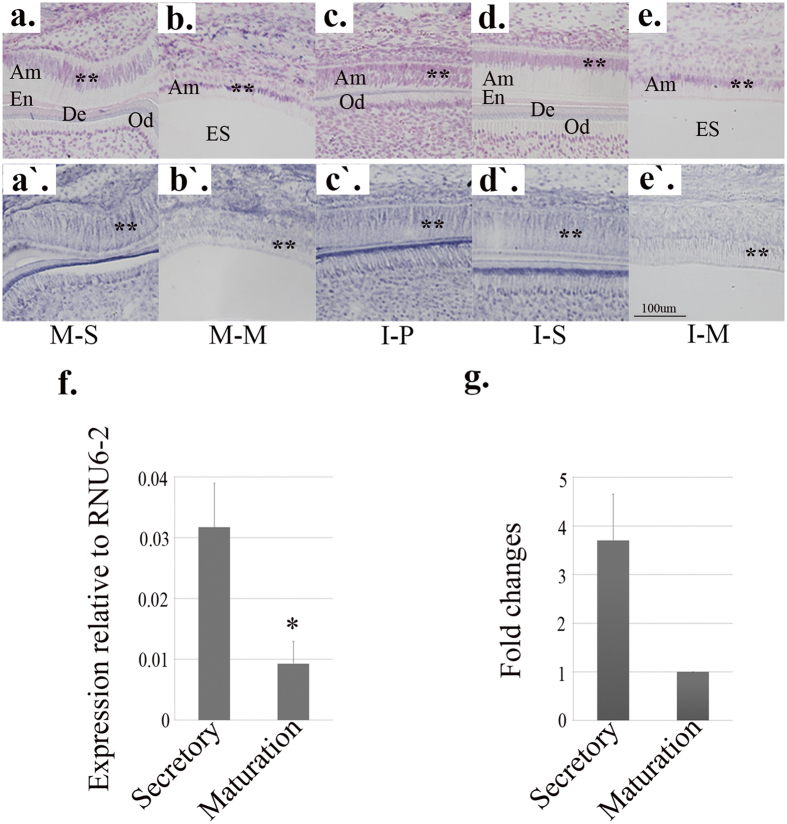
*In situ* hybridization and real-time PCR analysis of miR-153. Am: Ameloblast; En: Enamel; De: Dentin; Od: Odontoblast; ES: Enamel Space. M-S: Molar-Secretory stage; M-M: Molar-Maturation stage; I-P: Incisor-Presecretory stage; I-S: Incisor-Secretory stage; I-M: Incisor-Maturation stage. **Marks the signals from positive control (**a**–**e**) and miR-153 expression (**a**′–**e**′). (**a**,**a**′) secretory-stage enamel organ (PN6) of mouse mandibular first molar; (**b**,**b**′) maturation-stage enamel organ (PN9) of mouse mandibular first molar; (**c**,**c**′) presecretory-stage enamel organ of PN9 mouse mandibular incisor; (**d**,**d**′) secretory-stage enamel organ of PN9 mouse mandibular incisor; (**e**,**e**′) maturation-stage enamel organ of PN9 mouse mandibular incisor. Positive control for *in situ* hybridization is shown in panels (a–e) where the samples were incubated with U6 detection probes, and signals were mainly observed in the nuclei. MiR-153 expression by *in situ* hybridization is shown in panels a′–e′. The miR-153 signals developed from presecretory- and secretory-stage enamel organ (**a**′,**c**′,**d**′) were higher than those from maturation-stage enamel organ (**b**′,**e**′). The scale bar for all *in situ* hybridization images was 100 μm. Relative expression of miR-153 by real-time PCR using total RNAs isolated from secretory- and maturation-stage mandibular first molars of four animals (n = 4; panel f). The expression of miR-153 was significantly downregulated at maturation stage compared with secretory stage (*P = 0.002). The average fold change of miR-153 was ~−3.8 (maturation/secretory; panel g).

Commercially available LNA-DIG miRNA probes detected the expression patterns of miR-153 in presecretory-, secretory- and maturation-stage mouse enamel organs (Fig. 1a–e′). The morphologies of mouse enamel organs overlying mandibular first molars and incisors were first revisited by staining the PN6 and PN9 mandible section slides with U6 LNA-DIG probes (Fig. 1a–e), which also indicated a positive staining pattern of nuclei. *In situ* hybridization of miR-153 exhibited significantly higher signal intensities in presecretory- and secretory-stage enamel organs than in maturation-stage (Fig. 1a′–e′).

Using total RNA samples isolated from secretory-stage (PN6) and maturation-stage (PN9) mouse mandibular molars, the relative expression levels of miR-153 were quantified by real-time PCR analysis. Compared with secretory stage, miR-153 showed significantly decreased expression in maturation-stage enamel (*P* = 0.002, n = 4) (Fig. 1f); the fold change was approximately -3.8 (maturation/secretory, Fig. 1g). The results were consistent with the findings surfaced by *in situ* hybridization analysis (Fig. 1a′–e′).

### Endocytotic and endosomal/lysosomal pathways in maturation-stage enamel organ are marked by immunostaining

**Figure 2:**
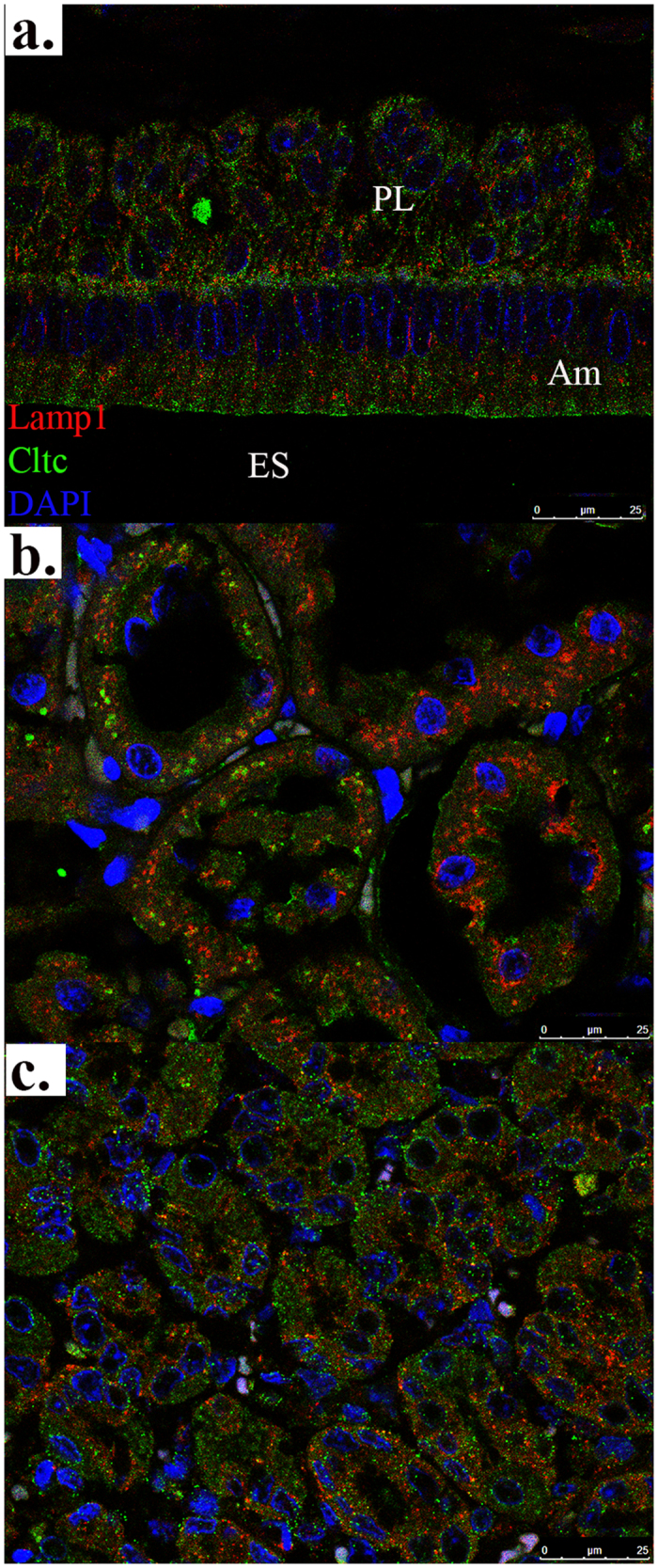
Endocytotic and endosomal/lysosomal pathways marked by immunofluorescence. Cltc and Lamp1 were marker genes for endocytotic and endosomal/lysosomal pathways in maturation-stage enamel organ overlying four-week-old rat mandibular incisor (**a**). The expression patterns of Cltc and Lamp1 in renal proximal tubules (**b**) and gastric submucosal glands (**c**) from four-week-old rat were stained as references. Red signals were from Lamp1 and green signals Cltc. All slides were counterstained by DAPI before cover slides were applied. The scale bar was 25 μm for all immunostaining images.

*Cltc* and *Lamp1* were used as marker genes for endocytotic and endosomal/lysosomal pathways in enamel organs^8^,^9^,^23^. In the maturation-stage enamel organ of 4-week-old rats, Cltc and Lamp1 were stained positively in both papillary layer^24^ and ameloblasts (Am) (Fig. 2a). Cltc in maturation-stage ameloblasts localized to the apical membrane and subapical cytoplasmic regions, while a main feature of Lamp1 expression was a peri-nuclear distribution pattern. In the cytoplasm halfway between apical membrane and nucleus, the staining from Cltc and Lamp1 showed some mixing and/or overlapping, which indicates the coupling of endocytotic and lysosomal pathways intracellularly (Fig. 2a). The expression patterns of Cltc and Lamp1 in renal proximal tubules (Fig. 2b) and gastric submucosal glands (Fig. 2c) were also shown by immunostaining as references.

### Selection strategies for miR-153 regulated gene targets

In the previous genome-wide miRNA and mRNA transcriptome analyses, miR-153 was identified as one of the differentially expressed miRNAs during amelogenesis in rats^11^. For the target prediction of miR-153, we combined the three algorithms integrated in Ingenuity Pathway Analysis (IPA, Ingenuity System, Redwood City, CA). The number of genes of interest was reduced by aligning the predicted genes with differentially expressed mRNAs from parallel mRNA microarray analysis, and a list of 993 annotated genes was generated. This group of predicted genes was uploaded to WebGestalt for Gene Ontology (GO) and KEGG analyses^25^,^26^,^27^ (Supplementary Fig. 1). By merging all the functional categories related to endocytotic and endosomal/lysosomal pathways, and arbitrarily setting a fold-change threshold at 3.0, the *Clcn4, Clcn5, Cltc, Ehd3, Lamp1, Lamp5, Rab7, Rab11fip2, Slc4a4, Slc26a7, Stam* and *Vps37a* genes were established as potential targets of miR-153 for further investigation.

### Intracellular miR-153 overloading induces changes in gene expression at miRNA, mRNA and protein levels

**Figure 3:**
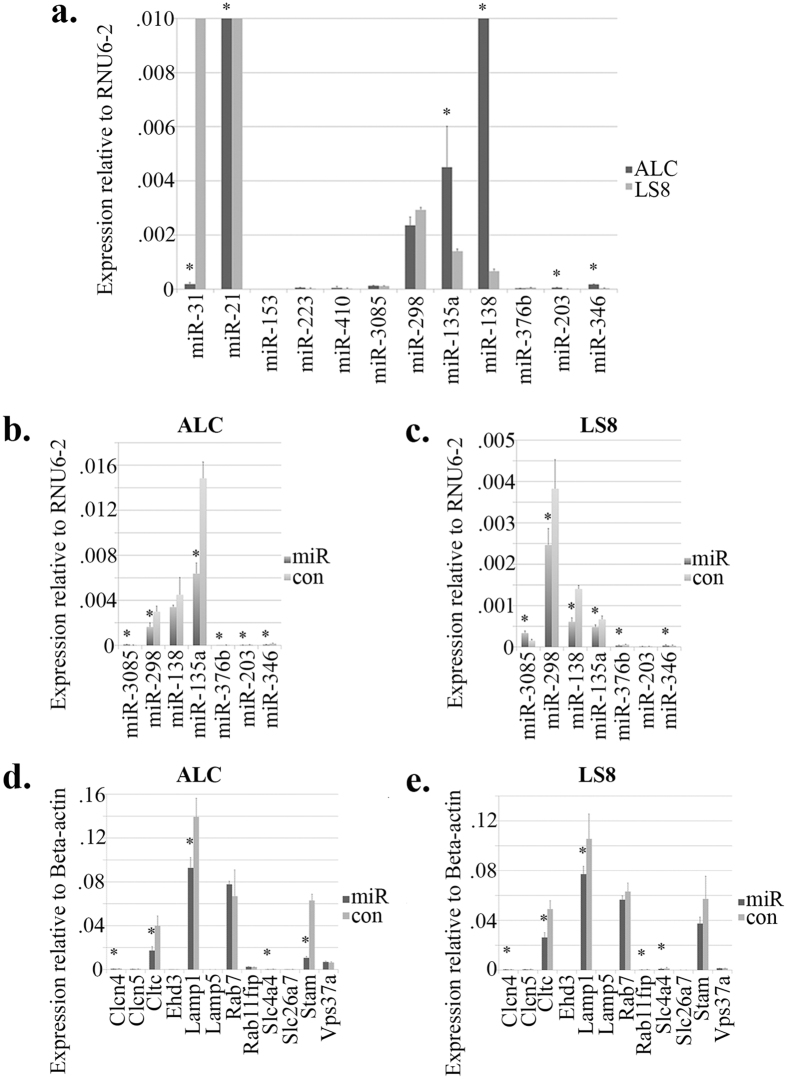
Baseline expression of miR-153 in ALCs and LS8 cells and MiR-153 induced changes in miRNA and mRNA expression by real-time PCR. (**a**) In both ALCs and LS8 cells, the expression of miR-153 was not detected by real-time PCR. MiR-31, miR-21, miR-135a, miR-138, miR-203 and miR-346 showed statistically significant differential expression between the two ameloblast-like cell models. The expression levels of miR-31, miR-21 and miR138 cannot be fully appreciated in the figure and the values are as follows: miR-31 in LS8 1.11 ± 0.0096, miR-21 in ALC 8.47 ± 0.94, miR-21 in LS8 1.35 ± 0.21, miR-138 in ALC 0.015 ± 0.0014. (**b**) MiRNA expression changes in ALCs. (**c**) MiRNA expression changes in LS8 cells. In miR-153 transfected ALCs compared with negative control siRNA transfected ALCs, the expression of miR-3085 and miR-346 was upregulated, while the expression of miR-298, miR-135a, miR-376b and miR-203 was downregulated. In LS8 cells, intracellular overloading of miR-153 induced similar changes in the expression of miR-3085, miR-298, miR-135a and miR-376b as those in ALCs. There were statistically significant differences in the expression of miR-138 and miR-346 in LS8 cells transfected by miR-153 and by negative control siRNA. (**d**) Gene expression changes in ALCs. (**e**) Gene expression changes in LS8 cells. Clcn4, Lamp1, Cltc and Slc4a4 exhibited significantly decreased expression in ALCs and LS8 cells transfected by miR-153. In ALCs, the expression of Stam was downregulated in miR-153 transfection group compared with mock transfection group. Rab11fip2 in miR-153 transfected LS8 cells showed increased expression. The expression of the remaining gene targets did not show any changes in response to miR-153 transfection in either cell model. The expression of Lamp5 was barely detected in both cells models. *P < 0.05.

For *in vitro* functional studies, ALCs and LS8 cells were used as hosts for miR-153. The baseline expression levels of miRNAs, which are predicted to be involved in regulating endocytosis and pH regulation^11^, were quantified by real-time PCR using RNA samples isolated from ALCs and LS8 cells without any treatment. The expression of miR-153 could not be detected in either cell model, which was in sharp contrast with the exceptionally high level of expression of miR-21 (Fig. 3a). Additionally, miR-31, miR-21, miR-135a, miR-138, miR-203 and miR-346 showed significantly differential expression patterns between ALCs and LS8 cells (*P* < 0.05) (Fig. 3a and Supplementary Table 1).

Intracellular miR-153 overloading was created by transiently transfecting ALCs and LS8 cells with miR-153 mimics. After 24 h of incubation following transfection, both cell models showed significant fluctuations in miRNA expression. Specifically, in miR-153 transfected ALCs, the expression of miR-3085 and miR-346 was upregulated compared with negative control siRNA, while the expression of miR-298, miR-135a, miR-376b and miR-203 was downregulated (*P* < 0.05) (Fig. 3b and Supplementary Table 1). In LS8 cells, the increased intracellular level of miR-153 induced similar directional changes (as in ALCs) in the expression of miR-3085, miR-298, miR-135a and miR-376b. In addition there were statistically significant differences in the expression of miR-138 and miR-346 in LS8 cells transfected by miR-153 and by negative control siRNA (*P* < 0.05) (Fig. 3c and Supplementary Table 1).

At mRNA level, the expression changes caused by miR-153 overloading were more consistent between the two cell models. Among all the gene targets, *Clcn4, Cltc, Lamp1* and *Slc4a4* exhibited significantly decreased expression in ALCs and LS8 cells transfected by miR-153 (*P* < 0.05) (Fig. 3d,e and Supplementary Table 1). In ALCs, the expression of *Stam* was significantly downregulated (Fig. 3d), while in LS8 cells, *Rab11fip2* also showed a significant decrease in expression. The remaining gene expression targets showed no significant changes in response to miR-153 transfection in either cell model. Note that miR-153 failed to trigger any changes in the mRNA-level expression of *Lamp5*, which was barely detectable in either cell model (Fig. 3d,e and Supplementary Table 1).

**Figure 4:**
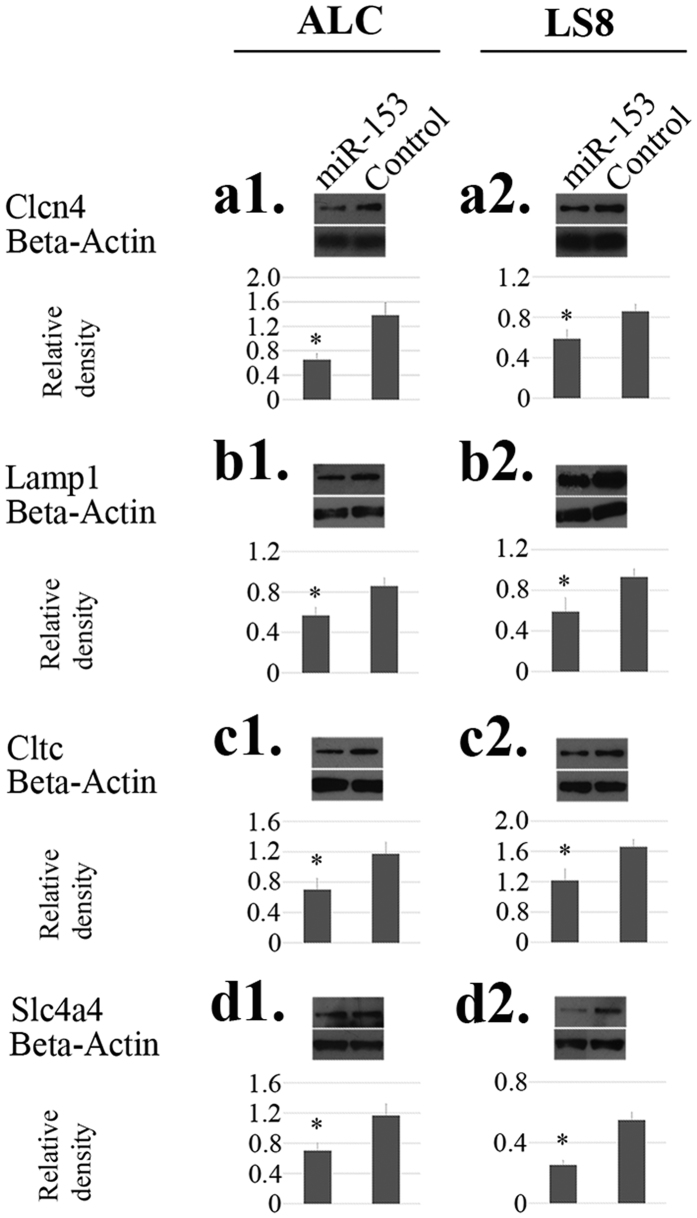
MiR-153 induced changes in gene expression at protein level by western blot. (**a1**–**d1**) Protein-level gene expression changes in ALCs. (**a2**–**d2**) Protein-level gene expression changes in LS8 cells. At protein level, intracellular overloading of miR-153 caused statistically significant downregulation of Clcn4 (**a1**,**a2**), Lamp1 (**b1**,**b2**), Cltc (**c1**,**c2**) and Slc4a4 (**d1**,**d2**), which corresponded well with the results from real-time PCR analysis at mRNA level. beta-actin served as controls for sample loading, and band densities were quantified relative beta-actin. *P < 0.05.

At the protein level, intracellular overloading of miR-153 caused statistically significant downregulation of Clcn4, Lamp1, Cltc and Slc4a4 (*P* < 0.05) (Fig. 4). The results correspond well with those generated from real-time PCR analysis at mRNA level (Fig. 3d,e and Supplementary Table 1).

### Cltc, Clcn4 and Slc4a4 are validated gene targets subject to miR-153 regulation

**Figure 5:**
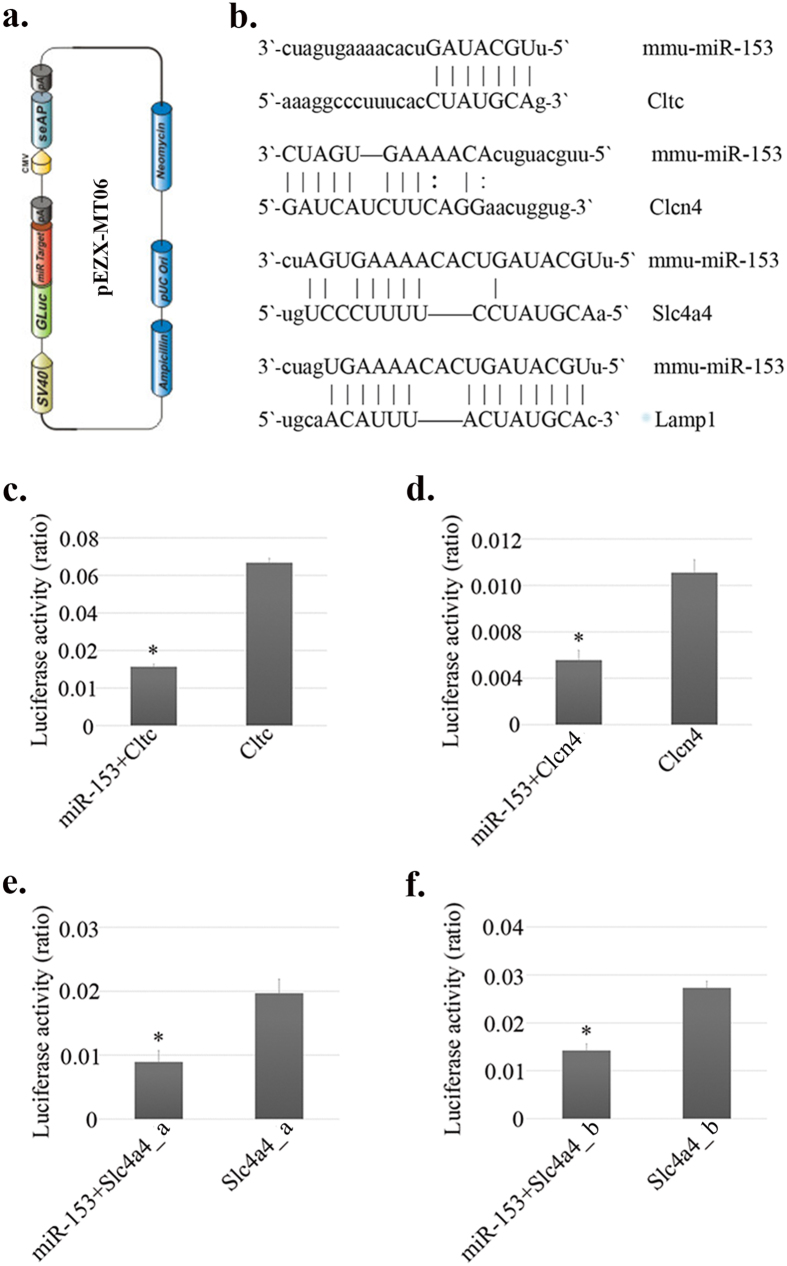
Luciferase reporter assay verifying the predicted interaction between miR-153 and target genes. (**a**) Luciferase vector construct. (**b**) Interactions between miR-153 and target genes predicted by TargetScan. Luciferase activities were presented in ratios of Renilla to Firefly luciferase reporter activities. In the luciferase reporter assays of Cltc (**c**) and Clcn4 (**d**), miR-153 mimics caused decreases of ~77% and ~46% in the luciferase reporter activities. The differences in luciferase reporter activities from Slc4a4 vectors (**e**,**f**) between miR-153 transfection group and mock transfection group were also statistically significant (a fragment: ~60% decrease in luciferase activity; b fragment: ~46% decrease in luciferase activity). *P < 0.05.

In the cell culture assays reported above, the intrinsic expression of Cltc, Clcn4, Slc4a4 and Lamp1 within ALCs and LS8 cells was suppressed by exogenously introduced miR-153 mimics at both mRNA and protein levels (Fig. 3b–e and Supplementary Table 1). The predicted interaction between miR-153 and the 3′-UTR of Lamp1 was validated previously by luciferase reporter assay^11^. As a result, three types of luciferase vectors containing the 3′-UTR of *Cltc, Clcn4* and *Slc4a4* were purchased for further investigation in this study (Genecopoeia). The vectors pertinent to *Slc4a4* came in two tubes containing an “a” and “b” fragments of 3′-UTRs, which corresponded with two different predicted interaction regions with miR-153. The more common predicted interaction (miR-153 with “a” fragment) was provided in Fig. 1, and the information about “b” fragment was not disclosed by the manufacturer. In the luciferase reporter assays of *Cltc* and *Clcn4*, miR-153 mimics caused decreases of ~77% and ~46% in the luciferase reporter activities (*P* = 0.012, *P* = 0.028) (Fig. 5c,d). The differences in luciferase reporter activities from *Slc4a4* vectors between miR-153 transfection group and mock transfection group were also found to be statistically significant (“a” fragment: ~60% decrease, *P* = 0.048; “b” fragment: ~46% decrease, *P* = 0.046) (Fig. 5e,f). Collectively, these data suggest that there were direct interactions between the seeding sequences of miR-153 and the 3′-UTRs of *Cltc, Clcn4* and *Slc4a4*.

### MiR-153 alters the response of ameloblast-like cells to extracellular EMPs

**Figure 6:**
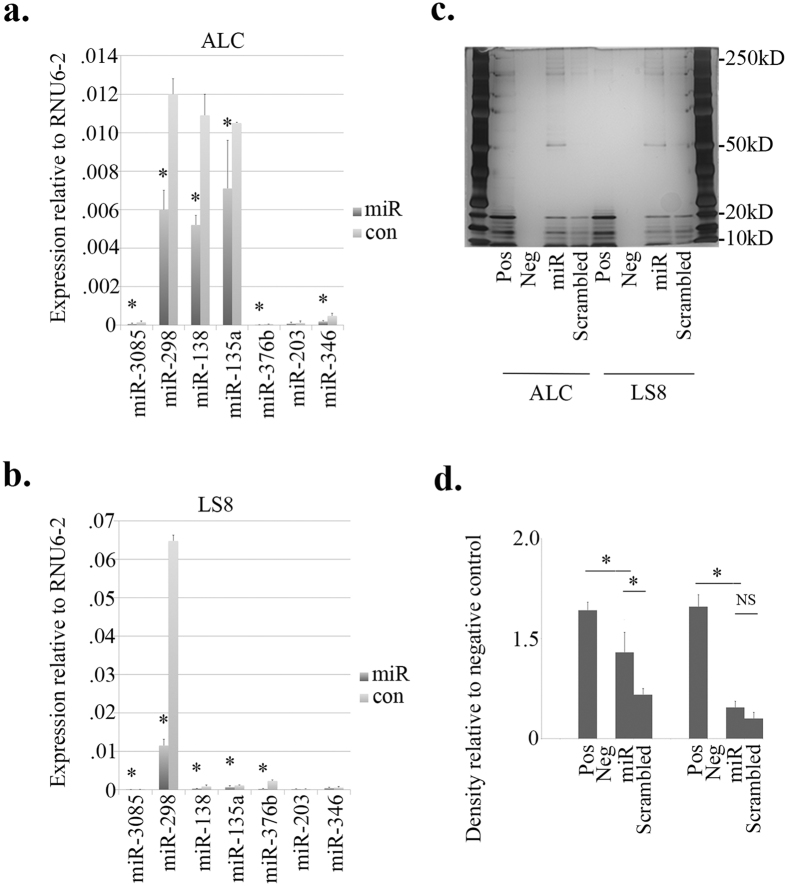
MiRNA expression changes caused by enamel matrix protein (EMD) exposure and EMD intake assay in presence of miR-153 overloading. There were two objectives of this experiment one was to evaluate the effect of miR-153 overloading on other miRNAs expressions after EMD exposure, and the second was to semi-quantify the effect of miR-153 overloading on EMD uptake. In both ALCs (**a**) and LS8 cells (**b**), treatment with EMD elicited significant downregulation of miR-3085, miR-298, miR-138, miR-135a and miR-376b. The ranges of miRNA expression fold changes in ALC cells were from ~−2.4 to −1.2 (**b**), and the fold changes of miRNAs in LS8 cells varied from ~−6.1 to −1.4 (**a**). The expression of miR-203 did not show any statistically significant changes in response to EMD uptake in either cell models (**a**,**b**), and decreased expression of miR-346 was only significant in ALCs (**a**). (**c**) After 6 h exposure to EMD, protein samples remaining in the medium of the cultured ALCs and LS8 cells were extracted for electrophoresis and silver staining. The presumable amelogenin bands are at ~20 kD. Positive control (Pos): no cells were seeded and EMD stock solution (2 mg/μl) was diluted to 500 μg/ml with FBS free culture medium; Negative control (Neg): enamel cells were seeded with no EMD treatment; MiR-153 transfection (miR): cells were transfected with miR-153 mimics and received treatment of EMD at 500 μg/ml for 6 h; Mock transfection with scrambled siRNA (Scrambled): cells were transfected with scrambled siRNAs and were exposed to 500 μg/ml EMD in the culture medium for 6 h. (**d**) 20 kD band densities were quantified relative to cell-free Positive control group. In ALCs and LS8 cells, miR-153 affected group showed significantly decreased relative band intensities compared with positive control group (ALC: ~−1.3-fold change positive/miR-153; LS8: ~−1.5-fold change positive/miR-153). When the miR-153 transfection group was compared with mock transfection group, the differences in relative band densities were determined to reach statistical significance only in ALCs (~−1.2-fold change scrambled/miR-153). *P < 0.05. NS Not significant.

Previous *in vitro* studies have demonstrated that LS8 cells take up fluorophore-labeled EMD^8^, and that the uptake of EMD leads to changes in gene transcription tied to clathrin-dependent endocytosis^23^. Here we applied EMD stock solution (2 mg/μl) to the culture medium of ALCs and LS8 cells to obtain a final concentration of 500 μg/ml. Following 6 h of exposure to EMD, RNA samples were isolated from cells transfected with miR-153 mimics and negative control siRNA for real-time PCR analysis of endocytosis-related miRNAs. Within both ALCs and LS8 cells, addition of EMD elicited significant downregulation of miR-3085, miR-298, miR-138, miR-135a and miR-376b (*P* < 0.05) (Fig. 6a,b and Supplementary Table 1). The fold changes of miRNAs in ALCs varied from ~−2.4 to −1.2, (Fig. 6a), while the fold changes of miRNA expression in LS8 cells varied from ~−6.1 to −1.4 (Fig. 6b). The expression of miR-203 did not show any statistically significant changes in response to EMD uptake in either cell model; and, decreased expression of miR-346 was only significant in ALCs (Fig. 6a,b and Supplementary Table 1).

With the purpose of evaluating the direct effect of miR-153 on cellular endocytotic functions, an additional EMD intake assay was performed. First, a series of EMD dilutions with concentrations ranging from 4.00 to 0.03 μg/μl were prepared by mixing the stock solution with FBS-free DMEM culture medium (Gibco^®^ Life Technologies). The EMD solutions were subjected to Tris-glycine gel electrophoresis and silver staining to generate referencing bands for subsequent analysis (Supplementary Fig. 2). For these series of experiments densitometry was used to quantitate the 20 kDa band only, which is the most abundant amelogenin product retrieved EMD^28^. Next, ALCs and LS8 cells were treated with extracellular EMD (at a final concentration of 500 μg/ml) for 6 h, the miR-153 affected group showed significantly decreased relative band intensities compared with the “no cell” positive control group (ALC: ~−1.3-fold change, *P* = 0.038; LS8: ~−1.5-fold change, *P* = 0.032) (Fig. 6c,d) indicating that both cell lines were resorbing/removing EMD from the medium. However, when the miR-153 transfection group was compared with the mock transfection group, the difference in relative band densities was statistically significant only in ALCs (~1.2-fold change, *P* = 0.040) (Fig. 6c,d). Although similar results evolved from the LS8 cells (i.e. greater resorption of the EMD was seen in the mock transfected group when compared to the miR-153 transfected group), the difference did not reach statistical significance (*P* < 0.05). In summary, this data shows that at-least in ALC cells miR-153 overloading results in a decrease in endocytotic activity.

### Local microinjection of miR-153 leads to hypomineralization of tooth enamel

**Figure 7:**
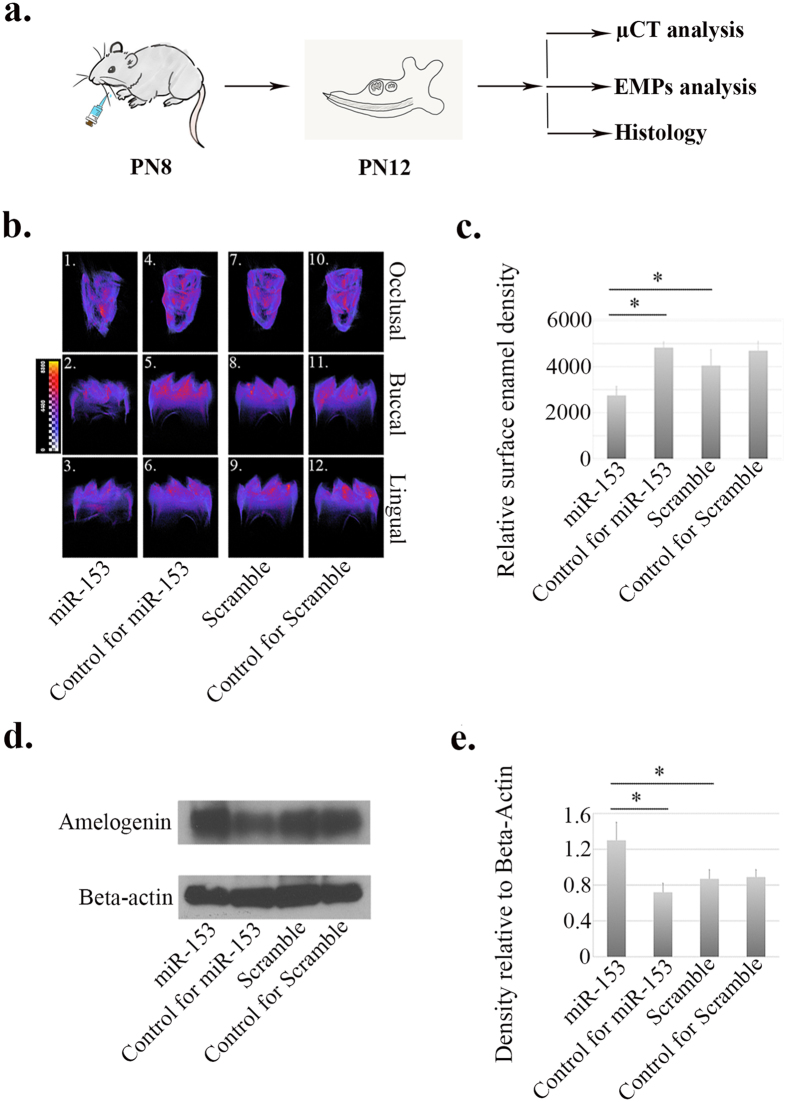
Microinjection assay. (**a**) Protocols for microinjection assay. (**b**,**c**) μCT analysis of miR-153 affected mandibular first molars. (**b**) 3-D reconstruction of PN12 mouse mandibular first molars. The colors varying from blue to red/yellow represents a change of enamel density from low to high. (**b1**–**b3**) miR-153: molars affected by miR-153 microinjection (right side mandibular injections); (**b4**–**b6**) Control for miR-153: left-side molars from the same animals as (**b1**–**b3**) with PBS (1X, pH 7.4) microinjection; (**b7**,**b8**) Scramble: molars affected by scrambled siRNA microinjection (right side mandibular injections); (**b10**–**b12**) Control for Scramble: left-side molar from (**b10**–**b12**) animals receiving PBS (1X, pH 7.4) microinjection. For the data presented, the concentration of miR-153 mimics and scrambled siRNA was 100 pmol. (**c**) Calculation and statistical analysis of relative surface enamel densities. When the miR-153 group was compared with PBS control group in the same animal, the potential differences in surface enamel densities reached statistical significance (*P* = 0.012). MiR-153 group showed an average of ~44% decrease in relative surface enamel density. The injection of scrambled siRNAs did not generate any statistically significant changes in surface enamel density relative to the PBS control side. Compared with scramble controls, the miR-153 affected mandibular first molars showed an average decrease of ~30% in relative enamel density (*P* = 0.034). (**d**,**e**) Western blot analysis of amelogenin in miR-153 injected mandibular first molars. (**d**) The target band of amelogenin was at 20-kD. Beta-actin was used to control the amount of sample loading. (**e**) The densities of target band were measured relative to beta-actin. Statistically significant differences were detected between miR-153 microinjection group (miR-153) and PBS control group (Control for miR-153), and between scrambled siRNA microinjection group (Scramble) and corresponding PBS control group (Control for scramble). *P < 0.05.

In the microinjection assay, we mainly sought to create an *in vivo* miR-153 overloading microenvironment and to evaluate its impact on maturation-stage enamel formation. Based on the previous findings, the expression of miR-153 is significantly downregulated at maturation-stage amelogenesis compared with secretory-stage (Fig. 1). As a result, we set PN8, at which the enamel organ of mouse mandibular first molar germs is generally at the end of secretory-stage amelogenesis, as the time point for the single microinjection of miR-153 mimics, and we sacrificed the animals at the end of maturation stage (PN12). Three concentrations of miR-153 were used; 10 pmol, 50 pmol and 100 pmol. Similar effects of miR-153 were observed in animals receiving either the 100 pmol and 50 pmol microinjection, while 10 pmol had no apparent impact on enamel development. Here, only data and our observations from the 100 pmol microinjection group are provided (Fig. 7). When the miR-153 treated PN12 mandibular first molars (panels A1–3) were compared with PBS controls (panels A4–6) in the same animal, the differences in surface enamel densities were found to be statistically significant (*P* = 0.012, Fig. 7b). To be more specific, exogenously introduced miR-153 caused an average of ~44% decrease in relative surface enamel density. On the other hand, the injection of scrambled siRNAs generated no statistically significant changes in surface enamel density (Fig. 7b), suggesting that the maturation-stage mineralization process was not disrupted by the injection procedure. Finally, compared with scramble controls, the miR-153 affected mandibular first molars showed an average decrease of ~30% in relative enamel density (*P* = 0.034, Fig. 7b), which indicated the causal relationship between miR-153 and enamel hypomineralization.

In order to investigate the mechanisms of miR-153 induced hypomineralization, protein samples were obtained from mandibular first molars that were subject to microinjection at PN8 and collected at PN12 for Western blot analysis. In miR-153 affected molars, an increased quantity of amelogenin was identified, compared with that in either PBS control side (~1.8-fold change, *P* = 0.023) or scramble controls (~1.4-fold change, *P* = 0.034) (Fig. 7c,d), which suggest that the AI-like signs caused by miR-153 microinjection may be attributed to the impaired function of ameloblasts to retrieve extracellular EMPs.

Despite the enamel density difference seen by μCT analyses, and the retained amelogenin protein seen in the same miR-153 treated mandibular molars, the morphology of the enamel organs overlying the mandibular first molars did not seem to be significantly influenced by miR-153/scrambled siRNA microinjection as observed through H&E staining of the PN12 mandibles (Supplementary Fig. 3).

## Discussion

Enamel formation is a mineralization process consisting of secretory and maturation stages. At secretory stage, there are four distinct layers of cells in the enamel organ: outer epithelium, stellate reticulum, stratum intermedium, and inner epithelium (also referred to as ameloblasts). The first three layers reorganize to form a papillary layer at the transition period from secretory to maturation stage^1^. Although the enamel organ contributes to the process of enamel development as an intact structure, most of the functional activities appear to be mediated by highly polarized ameloblasts that, at their apical pole, are in direct contact with the newly formed enamel structure. During maturation-stage tooth development, ameloblasts have been identified to be involved in a series of highly orchestrated events including matrix turnover, ion transport, pH regulation, calcium handling and cell apoptosis^2^,^10^. Previous studies have focused on the signaling pathways of enamel maturation primarily at genetic level^29^,^30^,^31^,^32^,^33^; however the involvement of epigenetic regulatory mechanisms in this process has not been widely studied.

**Figure 8:**
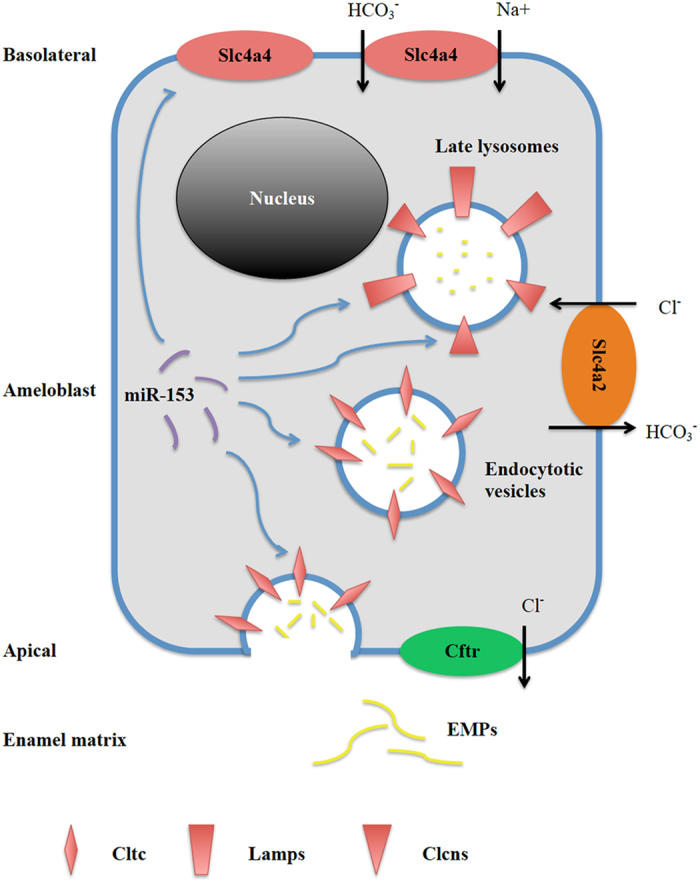
Schematic diagram for miR-153 regulated endocytotic and endosomal/lysosomal pathways. During maturation-stage tooth development, EMPs in the enamel matrix are broke down into protein fragments with smaller sizes. The EMP debris is retrieved by clathrin-coated vesicles, which fuse with lysosomes within ameloblast for further processing and degradation. MiR-153 targets endocytosis and lysosomal digestion by regulating the expression of Cltc, Lamp1, Clcn4 and Slc4a4. In the whole process of ameloblast-mediated removal of EMPs, the acid-base balance extracellularly and within intracellular lumens needs to be tightly controlled by various ion transporters/exchangers located on the apical and basolateral membrane of maturation-stage ameloblast.

We started to approach the issue by profiling genome-wide miRNA expression using RNA samples isolated from rat secretory- and maturation-stage enamel organs^11^. In our previous study^11^, we identified 59 differentially expressed miRNAs across the two stages of enamel formation. By aligning the list of predicted gene targets of miRNAs with the list of differentially expressed genes identified by parallel mRNA microarray analysis, we were able to scale down the number of predicted genes for miRNAs to ~600. The subsequent bioinformatic analysis of these predicted target genes highlighted functional categories that overlap with the major key processes in enamel maturation^11^. These data indicated that miRNAs not only exhibit a dynamic expression property during amelogenesis, but also actively participate in the regulatory networks of maturation-stage enamel formation. In the significantly enriched functional categories, such as those labeled with ‘endosome membrane’ or ‘lysosomal lumen’, miR-153 together with miR-3085, miR-298, miR-138, miR-135a, miR376b, miR-203 and miR-346 were predicted to be epigenetic regulators involved in endocytosis and endosomal/lysosomal pathways^11^. Furthermore, using luciferase reporter assay hosted by LS8 cells, miR-153 was confirmed to interact with the 3′-UTR of *Lamp1* which is one of the major genes in the process of EMP removal. As a result, the current study sought to investigate the miR-153-regulated endocytosis and endosomal/lysosomal pathways, and is a reasonable continuation of our previous effort to advance knowledge about miRNA-centered regulation during enamel maturation. We have presented a simplified schematic diagram to summarize the results (Fig. 8).

However, due to the intrinsic complexity of miRNA regulation, the investigation of one miRNA alone may not be able to cover all the details about an entire functional process. In the present study, we conducted both *in vitro* and *in vivo* functional studies and confirmed the involvement of miR-153 in the endocytosis and endosomal/lysosomal pathways by studying predicted miR-153 target genes. Although we used a relatively thorough searching strategy when screening the gene targets for miR-153, it is possible that there was a substantial false negative rate, which could be partially attributed to the limitations of available prediction algorithms. In addition, miR-153 is not the only miRNA regulator significantly enriched in the endocytotic and endosomal/lysosomal functional categories^11^. Exogenously introduced miR-153 caused significant upregulation and downregulation of these associated miRNA regulators (Fig. 3b,c), and the expression of many of these miRNAs responded to the addition of EMD into the medium of cultured enamel cells in the form of statistically significant downregulation (Fig. 6a,b). Thus, the role of this cluster of miRNAs and their predicted gene targets in enamel maturation still warrant further investigation.

As mentioned above, removal of EMPs is one of the key processes during maturation-stage amelogenesis^34^. Along with the presence of several proteinases in the enamel space to degrade EMPs^3^,^4^,^5^,^6^, the transporting mechanisms of EMP debris across ameloblasts are of equal importance in ensuring enamel health. Earlier understanding of cellular uptake of EMPs from enamel space seems to favor a passive diffusion or non-specific internalization pattern^12^,^13^,^14^,^15^,^16^. More recently, exogenously added fluorophore-labeled EMD was found to be rapidly mobilized to CD63/Lamp1-positive vesicles within LS8 cells suggesting an active (rather than passive) endocytotic pathway of ameloblasts exists for the removal of enamel matrix debris^8^. Using yeast-two hybrid system the specific protein regions with which Lamp1, Lamp2 and CD63 interact with EMPs has been shown, and such protein-protein interactions were believed to be involved in the removal of enamel protein debris and/or mediate signals to the transcriptional machinery^9^. More comprehensive studies, including data from a whole transcriptome analysis of maturation ameloblasts, identified the activities of more genes in EMP removal, and supported the existence of an AP-2 mediated, clathrin-dependent pathway in ameloblasts^7^,^10^. In our current study, miR-153 overloading impaired the ability of enamel cells to intake extracellular EMPs (Figs 6 and 7), which was achieved, at-least in part, by suppressing the expression of target genes—*Lamp1, Cltc, Clcn4* and *Slc4a4* (Figs 3, 4, 5). Collectively, these data provide evidence for the involvement of receptor-dependent pathways in the endocytosis of EMPs during enamel maturation.

In the process of bone formation, miRNAs play essential roles in regulating osteoblast differentiation, mineral deposition by the osteoblasts, and the osteoblast’s response to mechano-stimuli^35^,^36^,^37^,^38^,^39^. MiRNA intervention has been proposed as a candidate therapeutic modality to treat, or at least alleviate, skeletal disorders such as osteoporosis. Compared with bone mineralization, enamel formation possesses its own unique features and complexity. Nevertheless, more experimental evidence from miRNA regulated enamel formation is likely to deepen our understanding of the pathogenesis of enamel disorders. Among all the enamel diseases, AI is the most severe inherited disorder, which inflicts great suffering and treatment cost on patients. To date, identified genes responsible for AI include *AMELX, AMBN, ENAM, MMP20, KLK4, FAM83H* and *SLC24A4*; however, it is estimated that mutations in these genes account for less than half of the documented cases of AI^40^,^41^,^42^,^43^,^44^,^45^,^46^,^47^,^48^,^49^,^50^,^51^,^52^,^53^,^54^,^55^,^56^,^57^,^58^,^59^,^60^. Based on the present study, miR-153 overloading during enamel maturation induces significant hypomineralization—AI-like signs–in affected animal teeth (Fig. 7). This suggests that miR-153 overexpression at maturation-stage amelogenesis is a candidate etiologic factor in the occurrence of AI.

In conclusion, miRNAs are a group of dynamic regulators in the process of enamel formation^11^. Data in the present study indicate that miR-153 is actively involved in ameloblast-mediated endocytotic and endosomal/lysosomal pathways by directly targeting the key genes. New evidence from both *in vitro* and *in vivo* functional studies will advance our knowledge about miRNA-centered epigenetic regulation of amelogenesis, and provides novel insights into the pathogenesis of AI and potential preventative or therapeutic interventions.

## Methods

### Animal dissection and total RNA isolation

All vertebrate animal manipulation was carried out in accordance with Institutional and Federal guidelines. The animal protocols were approved by Institutional Animal Care and Use Committee at University of Southern California (Protocol# 11736). We used PN6 and PN9 mouse mandibular first molar as our source of total RNA, as these two time points correspond to the initiation of secretory- and maturation-stage enamel development, respectively. Four male wild-type BALB/c mice were sacrificed for their mandibular first molars. The extracted molars together with their surface epithelium from each animal were collected into separate RNase-free Eppendorf tubes containing QIAzol Lysis Reagent (Qiagen, Valencia, CA, USA). The total RNA including miRNA was isolated using miRNeasy Mini Kit (Qiagen). The total RNA obtained from the four mice was not pooled for real-time PCR analysis.

### *In situ* hybridization analysis of miR-153 expression

For miR-153 *in situ* hybridization, mandibles were dissected from PN6 and PN9 BALB/c mice, and the surrounding soft tissues were partially retained in order to keep the integrity of the mandibles. After being fixed in 4% paraformaldehyde (PFA) at 4 °C overnight, the PN6 and PN9 hemimandibles were decalcified in 10% EDTA (pH 7.4) for 10 days and 14 days, respectively. Sagittal sections of 7 μm were prepared from paraffin embedded tissue blocks. Commercially available miRNA probes (Exiqon, Inc., MA, USA) were used to detect miR-153 expression in enamel organs overlying mandibular first molars and incisors. Details regarding the procedures of miRNA *in situ* hybridization were described in previous studies^11^.

### Depicting endocytotic and endosomal/lysosomal pathways by immunofluorescence

Wistar Hannover rats (~100 g body weight, 4 weeks old) were sacrificed for their hemimandibles. The kidneys and stomach were also harvested as references. The hemimandibles were fixed in 4% PFA at 4 °C overnight and decalcified in 10% EDTA (pH 7.4) for 10–12 weeks. Seven μm tissue sections were dewaxed, rehydrated, blocked by 1% bovine serum albumin (BSA) in PBS (1X, pH 7.4) and incubated overnight with the primary antibodies against Lamp1 and Cltc (Supplementary Table 2)^8^,^9^,^23^. DAPI (Vector Laboratories; Catalog # H-1200) was applied as counterstaining before cover slides were added.

### Cell culture and transient transfection

Two lines of ameloblast-like mouse cells (ALCs and LS8 cells) were used as models for *in vitro* studies^61^. ALCs and LS8 cells were cultured in low-glucose DMEM medium (Gibco^®^ Life Technologies, Grand Island, NY, USA) with 10% Fetal Bovine Serum (FBS) at 37 °C in a 5% CO_2_ atmosphere. Freshly recovered cells were cultured for at least one week before any experiments were performed. The day before transfection, cells were seeded in 12-well cell culture plate to achieve approximately 30% confluency Lipofectamine^®^ LTX with plus^TM^ Reagent (Life Technologies) was diluted in FBS-free low-glucose DMEM medium in a concentration recommended by the manufacturers. MiR-153 mimics and negative control siRNA (Catalog # MSY0000163-miR-153 mimic, SI03650318-AllStars Negative Control siRNA, Qiagen) were mixed separately into diluted transfection reagents to form transfection complex. Immediately before transfection, the cell culture medium was changed into FBS-free low-glucose DMEM medium. This medium was removed 3 h after transfection, and cells were incubated in low-glucose DMEM medium with 10% FBS for 6 h, 24 h or 48 h, depending on the purpose of following experiments. The final concentrations tested for miR-153 and negative control siRNA are 20 pM, 60 pM and 120 pM.

### Real-time PCR analysis of cellular miRNA and mRNA levels

The RNA samples of first mandibular molars obtained from the four animals were converted to cDNA with miScript II RT Kit with miScript HiSpec Buffer (Qiagen). Real-time PCR reactions were performed with miScript SYBR Green PCR Kit (Qiagen) in CFX96 Touch™ Real-Time PCR Detection System (Bio-rad Life Science, Hercules, CA). Mouse-specific primers of miR-153 and RNA, U6 Small Nuclear 2 (RNU6-2) were purchased from Qiagen (Catalog # MS00011214-miR-153 primers, MS00033740-RNU6-2 primers). The expression levels of miR-153 in secretory- and maturation-stage enamel organs were calculated relative to the expression level of RNU6-2 as recommended by the manufacturer.

In order to identify that ALCs and LS8 cells are suitable models for investigating the functional role of miR-153, the baseline expression of miR-153 (along with miR-31, miR-21, miR-223, miR-410, miR-3085, miR-298, miR-135a, miR-138, miR376b, miR-203 and miR-346) was quantified by real-time PCR. The total RNA was isolated from ALCs and LS8 cells cultured in 12-well plates when confluency reached 100%. For quantification of miRNA and mRNA levels following miR-153 transfection, ALCs and LS8 cells were collected 24 h after transfection. Total RNA isolation, cDNA conversion and real-time PCR detection were performed as described above. The changes in miRNA and mRNA expression levels induced by miR-153 overloading were determined by comparing the miR-153 transfection group with a mock transfection group (transfected by AllStars Negative Control siRNA). The selection of miRNAs and mRNAs to be quantified is based on bioinformatic predictions^11^.

The information of other mouse-specific miRNA primers is as follows: Catalog # MS00001407-miR-31, MS00011487-miR-21, MS00032592-miR-223, MS00032823-miR-410, MS00025144-miR-3085, MS0000216-miR-298, MS00011130-miR-135a, MS00006041-miR-138, MS00002261-miR-376b, MS00001848-miR-203, MS00032753-miR-346 (Qiagen). The primers of Clcn4, Cltc, Edh3, Lamp1, Lamp5, Rab7, Slc4a4, Slc26a7, Stam and Vps37a were designed and synthesized by RealTimePrimers.com (www.realtimeprimers.com, Supplementary Table 3). The primers of Clcn5 and Rab11fip2 were designed by PrimerBank (pga.mgh.harvard.edu/primerbank), and synthesized by Invitrogen (Carlsbad, CA, USA, Supplementary Table 3).

### Western blot

Protein samples from miR-153 mimics and negative control siRNA transfected cells were obtained 48 h after transfection. RIPA Lysis and Extraction Buffer (Thermo Fisher Scientific Inc., Rockford, IL, USA; Catalog # 89901) mixed with Halt Protease Inhibitors Cocktail (Thermo Fisher Scientific; Catalog # 78429) was added into the 12-well cell culture plate (125 μl/well). Cells were detached from the bottom of the plate using scrapers of appropriate size. The collected samples were kept on ice for 30 min and centrifuged at 16,000 rpm for 15 min (4 °C) for supernatant. The protocols of Western blot analysis were similar to those previously described^62^. Information about antibody sources is provided in Supplementary Table 2.

### Luciferase reporter assay

LS8 cells were the hosts for miR-153 mimics and luciferase reporter vectors. Cell culture conditions and luciferase reporter assay protocols were as previously described^11^. Each luciferase reporter assay was conducted in triple technical replicates. Based on the results from real-time PCR and western blot following the miR-153 transfection assay, the potential targets of miR-153 were narrowed down to four genes–*Lamp1, Cltc, Clcn4* and *Slc4a4*. Because the interaction between the 3′-UTR of *Lamp1* and miR-153 was validated by luciferase reporter assay in previous studies^11^, three dual luciferase reporter vectors containing the 3′ UTR of mouse -specific target genes (*Cltc, Clcn4* and *Slc4a4*) were purchased from Genecopoeia (Catalog # MmiT073292-Cltc, MmiT023599-Clcn4, MmiT0301678-Slc4a4). For each verification assay, two experimental groups were set up: 1) LS8 cells co-transfected by miR-153 mimics and luciferase reporter vector (3′-UTR of target gene), 2) LS8 cells transfected by luciferase reporter vector (3′-UTR of target gene). The amount of luciferase reporter vector was stabilized at 700ng per transfection and the tested concentrations of miRNA mimics in the final transfection complex (after being added into FBS-free cell culture medium in 12-well plate) were 20 pM, 60 pM and 120 pM.

### Enamel matrix derivative (EMD) induced miRNA expression and intake assay

Lyophilized enamel matrix derivative or EMD (marketed as Emdogain; Straumann AG, Basel, Switzerland), composed primarily of porcine amelogenins^8^, was dissolved into 0.1% acetic acid to generate a stock solution of 2 mg/μl. ALCs and LS8 cells were seeded in 12-well cell culture plates at a confluency of 25%. After overnight incubation, the culture medium was replaced with FBS-free medium, the cells were carefully rinsed three times with sterilized PBS (pH 7.4) and transient transfection was then performed.

In the first part of the assay to examine changes in the expression of selected miRNAs, for each type of cell, the assay was conducted in two groups with each group taking up 3 wells: (1) miR-153 mimics transfection, (2) mock transfection with AllStars Negative Control siRNA. EMD stock solution was added into the cell culture medium to achieve a concentration of 500 μg/ml after another 24 h of incubation. Cells were maintained in standard culture conditions for 6 h (37 °C, 5% CO_2_), and the total RNA was extracted for miRNA expression quantification using real-time PCR.

In the second part of the assay to examine EMD uptake, there were four experimental groups (3 wells/group): (1) Positive control—no cells were seeded and EMD stock solution was simply mixed with FBS free culture medium, (2) Negative control—cells were seeded with no EMD stock solution being added, (3) miR-153 transfection—cells were transfected with miR-153 and EMD stock solution was applied in the culture medium to a final concentration of 500 μg/ml, (4) mock transfection—cells were transfected with AllStars Negative Control siRNA and EMD stock solution was added (to a final concentration of 500 μg/ml). For each experimental group, the cell-free culture medium was collected after 6 h of incubation. The samples (cell-free medium; 12 μl/well) were mixed with 4 x LDS Sample Loading Buffer (Thermo Fisher Scientific; Catalog # 84788), heated at 95 °C for 10 min, and loaded on mini gels (Thermo Fisher Scientific; Catalog # NP0315BOX) for electrophoresis (120 V, 2–2.5 h). The mini gels were stained with Pierce^TM^ Silver Stain Kit (Thermo Fisher Scientific; Catalog # 24612) for development of the protein bands from EMD. The protocols were as recommended by the manufacturer.

### Microinjection of miR-153 mimics

Animal-grade (*in vivo* application) miR-153 mimics (Qiagen; Catalog # MSY0000163) were dissolved into PBS (pH 7.4) (Thermo Fisher Scientific; Catalog # 10010023) to prepare final doses of 10 pmol, 50 pmol and 100 pmol. At PN8, each litter of wild-type BALB/c animals was randomly divided into two equal groups: (1) miR-153—animals were injected with miR-153 mimics, and (2) Negative control—animals were injected with AllStars Negative Control siRNAs. The location of microinjection (miR-153 mimics and negative control siRNAs, 10 μl per injection) was the right mandibular first molar germ area; the left side was injected with PBS as a control. At PN12, mandibles were dissected from sacrificed animals for hematoxylin and eosin (H&E) staining, and the mandibular first molars were extracted for μCT (SkyScan 1174, 50 kVp, 800 μA, 6.7 μm resolution) and Western blot analyses. The protocols of H&E staining and Western blot were similar to those mentioned in previous studies^23^,^62^. Each dosage level of microinjection was administered to three litters of animals.

### Statistical analysis

In the real-time PCR analysis of miR-153 expression level in enamel organs, the raw Ct values of miR-153 were normalized by those of RNU6-2. Fold changes (maturation/secretory) were calculated using the regular ΔΔCt method. The potential differences in the relative expression values of miR-153 between secretory and maturation stages were evaluated by two-tailed Student’s t-test (α = 0.05, SPSS 22.0). Similar data analysis strategies compared the baseline expression of miRNAs between ALCs and LS8 cells, and detected the changes in the cellular levels of miRNA and mRNA induced by miR-153 transient transfection. The expression values of mRNA were calculated relative to the reference gene beta-actin (Actb). The two-tailed Student’s t-test compared the difference in gene expression between the groups of miR-153 transfection and mock transfection.

Western blot analysis detected the protein-level changes of the target genes following miR-153 transfection, and the amount of remaining amelogenin in the mandibular first molar germ in microinjection assay. The relative densities of the target bands (normalized to the level of beta-actin) were quantified by NIH Image J software version 1.48. Because the sample size in Western blot analysis was relatively small, the Mann-Whitney U test was selected to detect the potential differences in protein-level gene expression due to miR-153 overloading both *in vitro* and *in vivo* (α = 0.05, SPSS 22.0). In the EMD intake assay, the relative band densities from silver stained gels were also quantified by Image J. All the values measured were presented relative to those in the negative control group. The differences in the relative densities between the positive control group and the miR-153 transfection group, and between the miR-153 transfection and mock transfection groups were detected by the Mann-Whitney U test (α = 0.05, SPSS 22.0).

Luciferase reporter assays were engaged to verify the predicted interaction between miR-153 and the 3′-UTR of its target genes. For each pair of measurements, *Renilla* luciferase activities were normalized by firefly luciferase activities to generate a ratio. Statistical differences in normalized luciferase activities between two experimental groups were detected by two-tailed Student’s t-test (α = 0.05, SPSS 22.0).

For μCT analysis of the mandibular first molar germs in microinjection assay, reconstruction and calculation of enamel density was performed with Amira 3D Visualization and Analysis Software 5.4.3 (FEI Visualization Science Group, Burlington, MA, USA). The two-tailed Student’s t-test was used to evaluate the potential differences in the relative enamel density between the miR-153 group and the scrambled siRNA group, in addition to the two-tailed paired t-test for the potential differences between the miR-153/scrambled siRNA injection side (right) and the control side (left) (α = 0.05, SPSS 22.0).

## Additional Information

**How to cite this article**: Yin, K. *et al*. MiR-153 Regulates Amelogenesis by Targeting Endocytotic and Endosomal/lysosomal Pathways–Novel Insight into the Origins of Enamel Pathologies. *Sci. Rep.* **7**, 44118; doi: 10.1038/srep44118 (2017).

**Publisher's note:** Springer Nature remains neutral with regard to jurisdictional claims in published maps and institutional affiliations.

## Supplementary Material

Supplementary Figure 1

Supplementary Figure 2

Supplementary Figure 3

Supplementary Table 1

Supplementary Table 2

Supplementary Table 3
