# Supplementary material for: MiR-153 Regulates Amelogenesis by Targeting Endocytotic and Endosomal/lysosomal Pathways–Novel Insight into the Origins of Enamel Pathologies
Source: Sci Rep. 2017 Mar 13;7:44118. doi: 10.1038/srep44118 (PMC5347039; doi:10.1038/srep44118)
Supplement: Supplementary Figure 1 [file srep44118-s1.pdf]

MiR-153 Regulates Amelogenesis by Targeting Endocytotic and Endosomal/lysosomal Pathways – Novel Insight into the Origins of Enamel Pathologies

Kaifeng Yin[1, 2], Wenting Lin[1], Jing Guo[3], Toshihiro Sugiyama[4], Malcolm L. Snead[1], Joseph G. Hacia[5], and Michael L. Paine[1]

[1] Center for Craniofacial Molecular Biology, Herman Ostrow School of Dentistry, University of Southern California, Los Angeles, CA, USA

[2] Department of Orthodontics, Herman Ostrow School of Dentistry, University of Southern California, Los Angeles, CA, USA

[3] Department of Endodontics, Herman Ostrow School of Dentistry, University of Southern California, Los Angeles, CA, USA

[4] Department of Biochemistry, Akita University of Graduate School of Medicine, Hondo, Akita, Japan

[5] Department of Biochemistry and Molecular Biology, Institute for Genetic Medicine, Keck School of Medicine, University of Southern California, Los Angeles, CA, USA

Supplementary fig 1. *MiR-153 regulated endocytotic and endosomal/lysosomal pathways generated by KEGG analysis*[25-27]. Genes labeled in red color were predicted targets for miR-153. The figures generated from KEGG have been granted permission by Kanehisa Laboratories to be used in this publication.

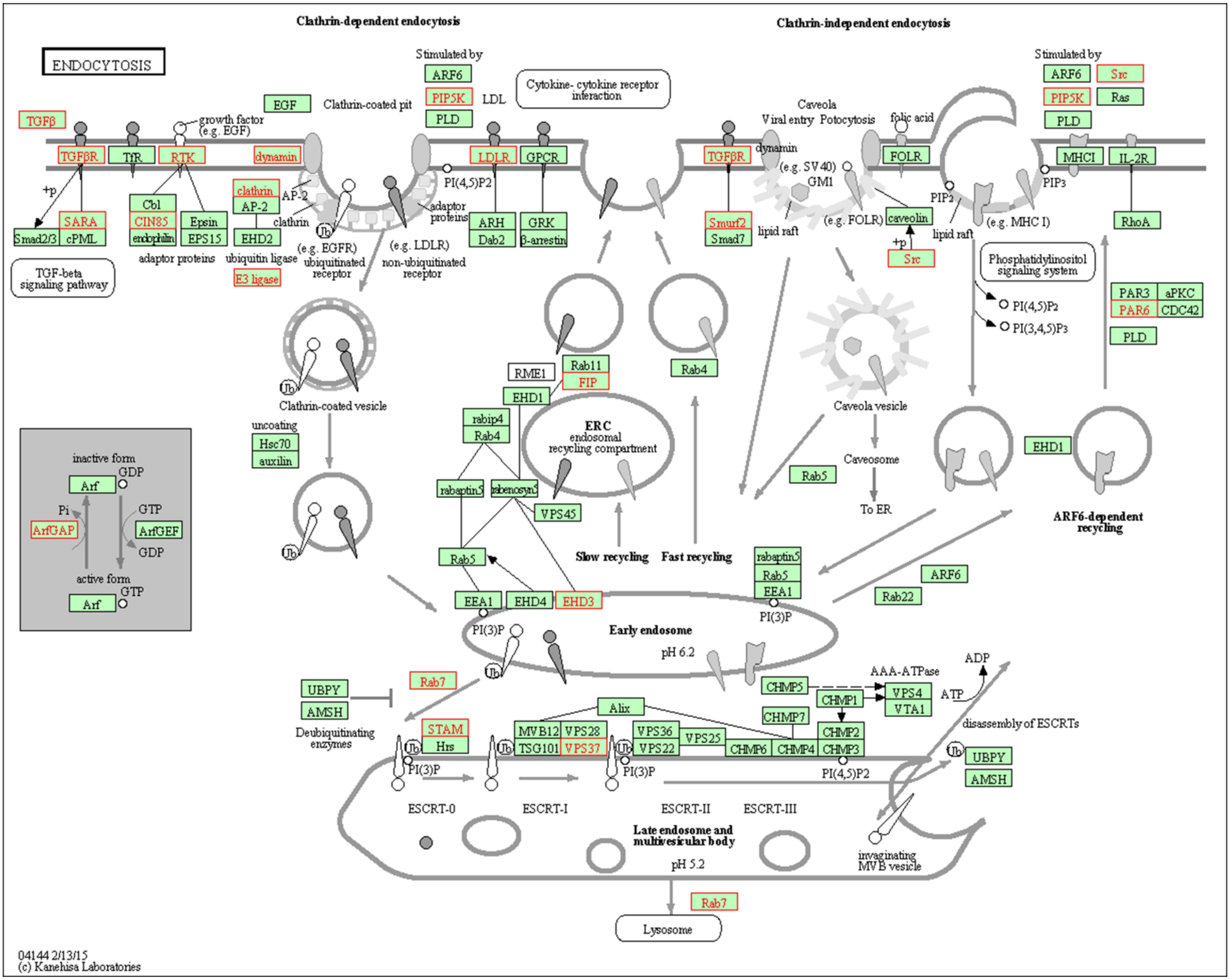
Clathrin-dependent endocytosis
Clathrin-independent endocytosis
ENDOCYTOSIS
Stimulated by
ARF6
PIP5K
PLD
Cytokine- cytokine receptor interaction
EGF
Clathrin-coated pit
LDL
TGFβ
growth factor (e.g. EGF)
TGFβR
TfR
RTK
dynamin
LDLR
GPCR
PI(4,5)P2
+p
SARA
Smad2/3
cPML
Cbl
CIN85
endophilin
Epsin
EPS15
adaptor proteins
clathrin
AP-2
EHD2
ubiquitin ligase
E3 ligase
(e.g. EGFR) ubiquitinated receptor
(e.g. LDLR) non-ubiquitinated receptor
adaptor proteins
ARH
Dab2
GRK
β-arrestin
TGF-beta signaling pathway
Clathrin-coated vesicle
uncoating
Hsc70
auxilin
inactive form
GDP
Arf
Pi
GTP
ArfGAP
ArfGEF
active form
Rab11
FIP
RME1
EHD1
rabip4
Rab4
ERC endosomal recycling compartment
rabaptin5
rabenosyn5
VPS45
Rab5
Slow recycling
Fast recycling
EEA1
EHD4
EHD3
Early endosome
PI(3)P
pH 6.2
Caveola
Viral entry
Potocytosis
folic acid
FOLR
TGFβR
Smurf2
Smad7
(e.g. SV40)
GM1
lipid raft
(e.g. FOLR)
caveolin
Src
Caveola vesicle
Caveosome
To ER
Stimulated by
Src
Ras
MHCI
IL-2R
PIP2
PIP3
(e.g. MHC I)
RhoA
Phosphatidylinositol signaling system
PI(4,5)P2
PI(3,4,5)P3
PAR3
aPKC
PAR6
CDC42
PLD
EHD1
ARF6-dependent recycling
ARF6
Rab22
UBPY
AMSH
Deubiquitinating enzymes
Rab7
STAM
Hrs
Alix
MVB12
VPS28
TSG101
VPS37
VPS36
VPS22
VPS25
CHMP6
CHMP4
CHMP3
CHMP2
CHMP5
CHMP1
CHMP7
VPS4
VTA1
AAA-ATPase
ATP
ADP
disassembly of ESCRTs
ESCRT-0
ESCRT-I
ESCRT-II
ESCRT-III
PI(4,5)P2
Late endosome and multivesicular body
pH 5.2
invaginating MVB vesicle
Lysosome
04144 2/13/15
(c) Kanehisa Laboratories
