# Supplementary material for: MiR-153 Regulates Amelogenesis by Targeting Endocytotic and Endosomal/lysosomal Pathways–Novel Insight into the Origins of Enamel Pathologies
Source: Sci Rep. 2017 Mar 13;7:44118. doi: 10.1038/srep44118 (PMC5347039; doi:10.1038/srep44118)
Supplement: Supplementary Figure 2 [file srep44118-s2.pdf]

MiR-153 Regulates Amelogenesis by Targeting Endocytotic and Endosomal/lysosomal Pathways – Novel Insight into the Origins of Enamel Pathologies

Kaifeng Yin[1, 2], Wenting Lin[1], Jing Guo[3], Toshihiro Sugiyama[4], Malcolm L. Snead[1], Joseph G. Hacia[5], and Michael L. Paine[1]

[1] Center for Craniofacial Molecular Biology, Herman Ostrow School of Dentistry, University of Southern California, Los Angeles, CA, USA

[2] Department of Orthodontics, Herman Ostrow School of Dentistry, University of Southern California, Los Angeles, CA, USA

[3] Department of Endodontics, Herman Ostrow School of Dentistry, University of Southern California, Los Angeles, CA, USA

[4] Department of Biochemistry, Akita University of Graduate School of Medicine, Hondo, Akita, Japan

[5] Department of Biochemistry and Molecular Biology, Institute for Genetic Medicine, Keck School of Medicine, University of Southern California, Los Angeles, CA, USA

Supplementary fig 2. *Silver staining of EMD on Tris-glycine minigel.* EMD solutions with the concentration of 4.00 to 0.03 μg/μl were subject to Tris-glycine minigel electrophoresis. The series of presumably amelogenin bands (~20kD) developed by silver staining served as references for subsequent gel staining with the same method.

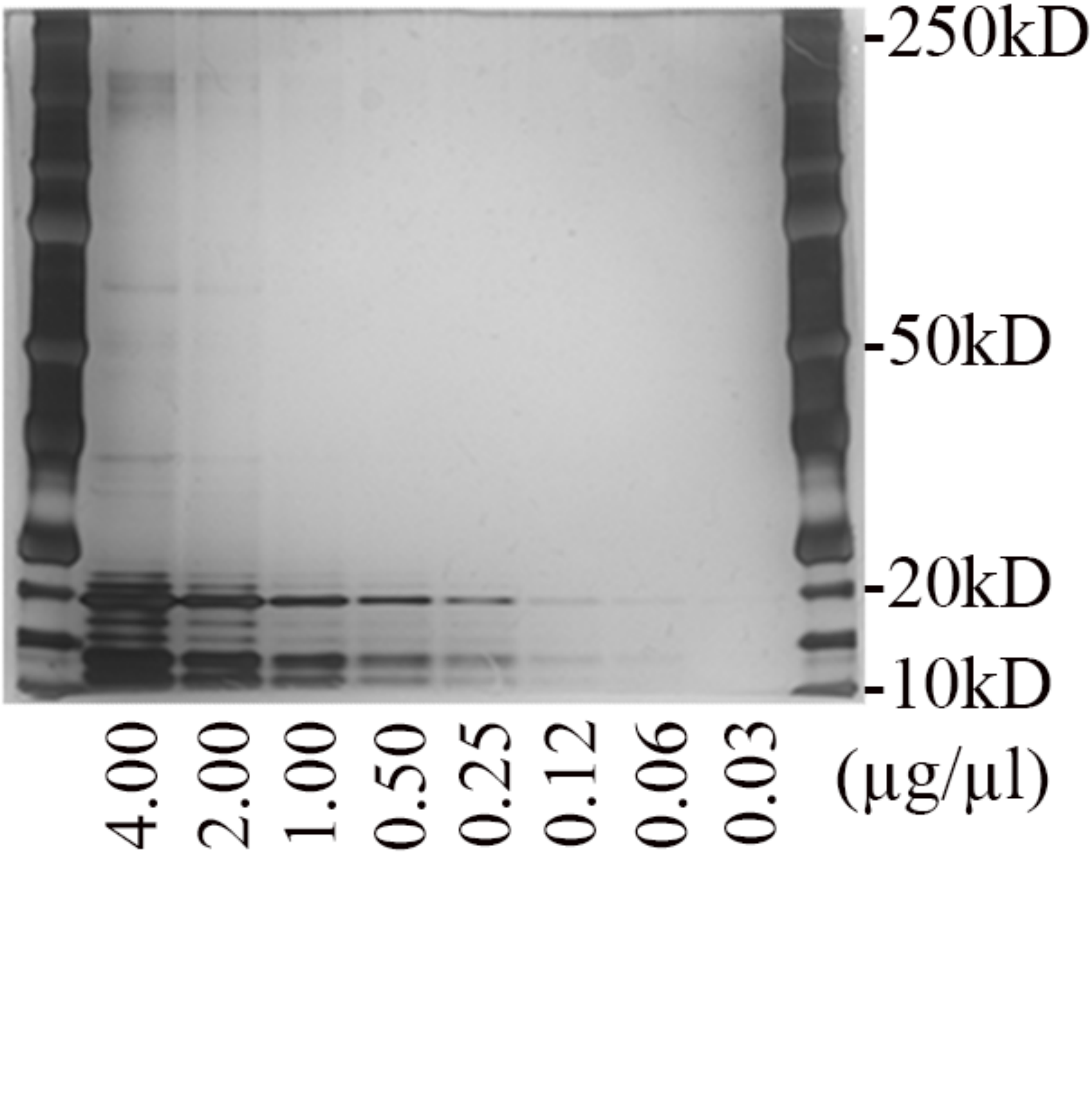
-250kD
-50kD
-20kD
-10kD
4.00
2.00
1.00
0.50
0.25
0.12
0.06
0.03
(μg/μl)
