# Supplementary material for: MiR-153 Regulates Amelogenesis by Targeting Endocytotic and Endosomal/lysosomal Pathways–Novel Insight into the Origins of Enamel Pathologies
Source: Sci Rep. 2017 Mar 13;7:44118. doi: 10.1038/srep44118 (PMC5347039; doi:10.1038/srep44118)
Supplement: Supplementary Figure 3 [file srep44118-s3.pdf]

# MiR-153 Regulates Amelogenesis by Targeting Endocytotic and Endosomal/lysosomal Pathways – Novel Insight into the Origins of Enamel Pathologies

Kaifeng Yin[1, 2], Wenting Lin[1], Jing Guo[3], Toshihiro Sugiyama[4], Malcolm L. Snead[1], Joseph G. Hacia[5], and Michael L. Paine[1]

[1] Center for Craniofacial Molecular Biology, Herman Ostrow School of Dentistry, University of Southern California, Los Angeles, CA, USA

[2] Department of Orthodontics, Herman Ostrow School of Dentistry, University of Southern California, Los Angeles, CA, USA

[3] Department of Endodontics, Herman Ostrow School of Dentistry, University of Southern California, Los Angeles, CA, USA

[4] Department of Biochemistry, Akita University of Graduate School of Medicine, Hondo, Akita, Japan

[5] Department of Biochemistry and Molecular Biology, Institute for Genetic Medicine, Keck School of Medicine, University of Southern California, Los Angeles, CA, USA

**Supplementary fig 3.** *H&E staining of enamel organs of PN12 mandibles from miR-153 microinjection assay.* (a) MiR-153 microinjection group (right side mandibular injection); (b) PBS control group for miR-153 microinjection (left side mandibular injection); (c) Scrambled siRNA microinjection groups (right side mandibular injection); (d) PBS control group for scrambled siRNA microinjection (left side mandibular injection). ** signifies the location of enamel organ. The morphology of maturation-stage enamel organs did not show any differences across different treatment groups.

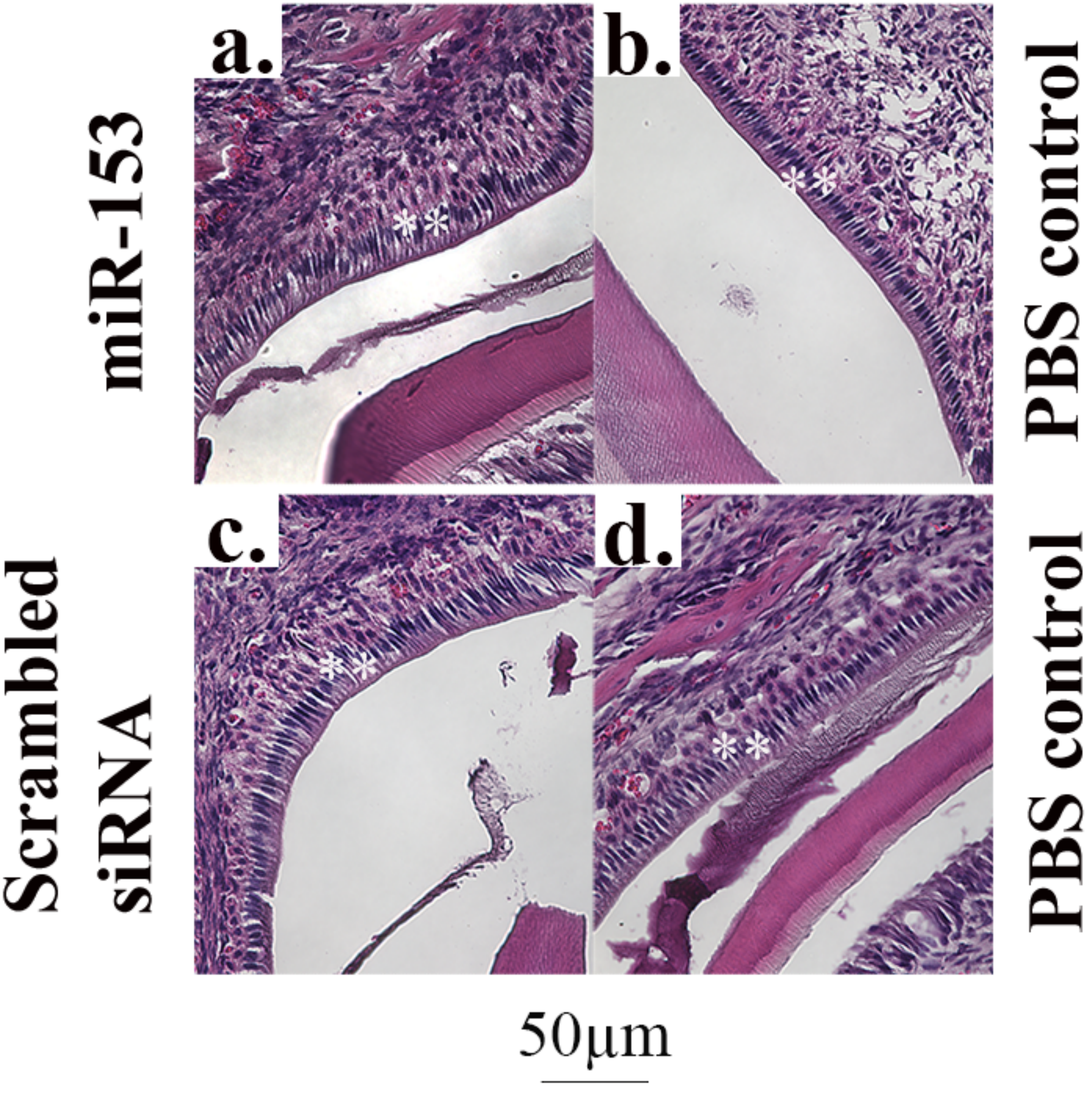

a.
b.
c.
d.
miR-153
PBS control
Scrambled siRNA
PBS control
50μm
