# Supplementary material for: MiR-153 Regulates Amelogenesis by Targeting Endocytotic and Endosomal/lysosomal Pathways–Novel Insight into the Origins of Enamel Pathologies
Source: Sci Rep. 2017 Mar 13;7:44118. doi: 10.1038/srep44118 (PMC5347039; doi:10.1038/srep44118)
Supplement: Supplementary Table 1 [file srep44118-s4.pdf]

MiR-153 Regulates Amelogenesis by Targeting Endocytotic and Endosomal/lysosomal Pathways – Novel Insight into the Origins of Enamel Pathologies

Kaifeng Yin[1, 2], Wenting Lin[1], Jing Guo[3], Toshihiro Sugiyama[4], Malcolm L. Snead[1], Joseph G. Hacia[5], and Michael L. Paine[1]

[1] Center for Craniofacial Molecular Biology, Herman Ostrow School of Dentistry, University of Southern California, Los Angeles, CA, USA

[2] Department of Orthodontics, Herman Ostrow School of Dentistry, University of Southern California, Los Angeles, CA, USA

[3] Department of Endodontics, Herman Ostrow School of Dentistry, University of Southern California, Los Angeles, CA, USA

[4] Department of Biochemistry, Akita University of Graduate School of Medicine, Hondo, Akita, Japan

[5] Department of Biochemistry and Molecular Biology, Institute for Genetic Medicine, Keck School of Medicine, University of Southern California, Los Angeles, CA, USA

Supplementary Table 1. Raw data for qPCR analyses (Figs 3 and 6)

Fig 3a

Gene symbol	Relative expression value (ALC/LS8)	Standard deviation (ALC/LS8)
miR-31	0.000187/1.11 *	6.22e-05/0.0959
miR-21	8.47/1.35 *	0.937/0.208
miR-153	n/a	n/a
miR-223	5.15/2.08e-05	1.33e-05/2.00e-05
miR-410	4.72-e05/1.83e-05	5.00-e05/1.92-e05
miR-3085	0.000128/0.000109	1.53e-05/1.64e-05
miR-298	0.00235/0.00293	0.000317/8.94e-05
miR-135a	0.00450/0.00140 *	0.00152/8.40e-05
miR-138	0.0148/0.000666 *	0.00146/8.06e-05
miR-376b	2.67e-05/5.92e-05	8.52e-05/2.39e-07
miR-203	5.81e-05/1.04e-05 *	9.22e-05/2.01e-06
miR-346	0.000176/3.46e-05 *	1.05e-05/1.04e-05

Fig 3b

Gene symbol	Relative expression value (miR/con)	Standard deviation (miR/con)
miR-3085	8.62e-05/2.81e-05 *	3.31e-06/5.10e-06
miR-298	0.00163/0.00299 *	0.000350/0.0005
miR-138	0.00339/0.00450	0.000170/0.00152
miR-135a	0.00637/0.0148 *	0.000952/0.00146
miR-376b	5.19e-06/2.67e-05 *	4.59e-07/8.52e-06
miR-203	3.47e-05/5.81e-05 *	7.34e-06/9.22e-06
miR-346	0.00017558/6.88e-05 *	1.55e-05/1.05e-05

Fig 3c

Gene symbol	Relative expression value (miR/con)	Standard deviation (miR/con)
miR-3085	0.000332/0.000150 *	5.17e-05/3.14e-05
miR-298	0.00246/0.00383 *	0.000399/0.000701
miR-138	0.000608/0.00140 *	9.27e-05/8.40e-05
miR-135a	0.000481/0.000666 *	5.27e-05/8.06e-05
miR-376b	4.09e-05/5.92e-05 *	2.99e-06/2.39e-07
miR-203	1.02e-05/1.04e-05	2.01e-06/1.87e-06
miR-346	3.93e-05/3.46e-05 *	1.04e-05/1.04-e-05

Fig 3d

Gene symbol	Relative expression value (miR/con)	Standard deviation (miR/con)
Clcn4	0.000523/0.000685 *	8.09e-05/1.09e-05
Clcn5	0.000414/0.000463	4.72e-05/0.000111
Cltc	0.0172/0.0398 *	0.00377/0.00901

Ehd3	2.02e-06/3.02e-06	5.37e-07/1.36e-06
Lamp1	0.0928/0.139 *	0.00939/0.0168
Lamp5	n/a	n/a
Rab7	0.0776/0.0668	0.00300/0.0241
Rab11fip2	0.00234/0.00221	0.000434/0.000109
Slc4a4	0.000192/0.000308 *	3.97e-05/2.62e-05
Slc26a7	4.88e-05/9.95e-05	1.94e-05/3.57e-05
Stam	0.0106/0.0630 *	0.00128/0.00577
Vps37a	0.00697/0.00641	0.000676/0.000680

Fig 3e

Gene symbol	Relative expression value (miR/con)	Standard deviation (miR/con)
Clcn4	0.000343/0.000450 *	6.61e-05/1.07e-05
Clcn5	0.000444/0.000542	8.90e-05/0.000139
Cltc	0.0261/0.0490 *	0.0393/0.00681
Ehd3	2.43e-06/2.25e-06	5.98e-07/5.38e-07
Lamp1	0.077/0.106 *	0.00647/0.020
Lamp5	n/a	n/a
Rab7	0.0566/0.0630	0.00317/0.00704
Rab11fip2	0.000225/0.000478 *	4.92e-05/0.000124
Slc4a4	0.000841/0.00129 *	0.000629/0.000916
Slc26a7	6.19e-05/8.44e-05	2.09e-05/2.07e-05
Stam	0.0373/0.0572	0.00529/0.00182
Vps37a	0.00137/0.00148	0.000194/5.14e-05

Fig 6a

Gene symbol	Relative expression value (miR/con)	Standard deviation (miR/con)
miR-3085	6.02e-05/0.000150 *	4.10e-05/7.42e-05
miR-298	0.00651/0.0122 *	0.00107/0.000805
miR-138	0.00520/0.0109 *	0.000561/0.00110
miR-135a	0.00714/0.0105 *	0.00259/4.09e-05
miR-376b	1.89e-05/5.65e-05 *	4.79e-06/1.17e-06
miR-203	8.98e-05/0.000110	7.24e-05/0.0000980
miR-346	0.000197/0.000483 *	3.92e-05/0.00013

Fig 6b

Gene symbol	Relative expression value (miR/con)	Standard deviation (miR/con)
miR-3085	6.70e-05/9.12e-05 *	5.67e-06/4.58e-06
miR-298	0.0115/0.0648 *	0.00163/0.00152
miR-138	0.000314/0.000865 *	1.78e-05/0.000344
miR-135a	0.000690/0.00113 *	0.000304/0.000132
miR-376b	0.000203/0.0023 *	8.45e-05/0.000278
miR-203	0.000133/0.000111	8.71e-05/9.90e-05

miR-346	0.000446/0.000559 *	0.000212/0.000241
