# Supplementary material for: MiR-153 Regulates Amelogenesis by Targeting Endocytotic and Endosomal/lysosomal Pathways–Novel Insight into the Origins of Enamel Pathologies
Source: Sci Rep. 2017 Mar 13;7:44118. doi: 10.1038/srep44118 (PMC5347039; doi:10.1038/srep44118)
Supplement: Supplementary Table 2 [file srep44118-s5.pdf]

MiR-153 Regulates Amelogenesis by Targeting Endocytotic and Endosomal/lysosomal Pathways – Novel Insight into the Origins of Enamel Pathologies

Kaifeng Yin[1, 2], Wenting Lin[1], Jing Guo[3], Toshihiro Sugiyama[4], Malcolm L. Snead[1], Joseph G. Hacia[5], and Michael L. Paine[1]

[1] Center for Craniofacial Molecular Biology, Herman Ostrow School of Dentistry, University of Southern California, Los Angeles, CA, USA

[2] Department of Orthodontics, Herman Ostrow School of Dentistry, University of Southern California, Los Angeles, CA, USA

[3] Department of Endodontics, Herman Ostrow School of Dentistry, University of Southern California, Los Angeles, CA, USA

[4] Department of Biochemistry, Akita University of Graduate School of Medicine, Hondo, Akita, Japan

[5] Department of Biochemistry and Molecular Biology, Institute for Genetic Medicine, Keck School of Medicine, University of Southern California, Los Angeles, CA, USA

Supplementary Table 2. Antibodies used for immunofluorescence and western blot

Gene product detected	Manufacturer	Application(s)
Lamp1	Abcam (ab24170)	Immunofluorescence (dilution 1:400)<br>Western blot (dilution 1:1000)
Cltc	Abcam (ab2731)	Immunofluorescence (dilution 1:500)
Cltc	Abcam (ab21679)	Western blot (dilution 1:1000)
Clcn4	Abcam (ab75008)	Western blot (dilution 1:500)
Slc4a4	Santa Cruz Biotechnology (sc-162214)	Western blot (dilution 1:200)
Beta Actin	Abcam (ab6276)	Western blot (dilution 1:3000)
