# Supplementary material for: MiR-153 Regulates Amelogenesis by Targeting Endocytotic and Endosomal/lysosomal Pathways–Novel Insight into the Origins of Enamel Pathologies
Source: Sci Rep. 2017 Mar 13;7:44118. doi: 10.1038/srep44118 (PMC5347039; doi:10.1038/srep44118)
Supplement: Supplementary Table 3 [file srep44118-s6.pdf]

MiR-153 Regulates Amelogenesis by Targeting Endocytotic and Endosomal/lysosomal Pathways – Novel Insight into the Origins of Enamel Pathologies

Kaifeng Yin[1, 2], Wenting Lin[1], Jing Guo[3], Toshihiro Sugiyama[4], Malcolm L. Snead[1], Joseph G. Hacia[5], and Michael L. Paine[1]

[1] Center for Craniofacial Molecular Biology, Herman Ostrow School of Dentistry, University of Southern California, Los Angeles, CA, USA

[2] Department of Orthodontics, Herman Ostrow School of Dentistry, University of Southern California, Los Angeles, CA, USA

[3] Department of Endodontics, Herman Ostrow School of Dentistry, University of Southern California, Los Angeles, CA, USA

[4] Department of Biochemistry, Akita University of Graduate School of Medicine, Hondo, Akita, Japan

[5] Department of Biochemistry and Molecular Biology, Institute for Genetic Medicine, Keck School of Medicine, University of Southern California, Los Angeles, CA, USA

Supplementary Table 3. Mouse-specific primers for qPCR and cDNA analyses

Symbol	Accession	Size	Region	Forward (5`-3`)<br>Reverse (5`-3`)
Actb	NM_007393	160	792-951	AAGAGCTATGAGCTGCCTGA<br>TACGGATGTCAACGTCACAC
Clcn4	NM_011334	150	552-701	TACGAGGACTTCCACACCAT<br>GAGTAGCATCACCACCCATC
Clcn5	NM_016691	101	223-323	GAGGAGCCAATCCCTGGTGTA<br>TTGGTAATCTCTCGGTGCCTA
Cltc	NM_001003908	193	3693-3885	GCTCGAGAGTCCTATGTGGA<br>CCAAGCGTCCAAAGTTAGAA
Edh3	NM_020578	203	219-421	ACACAATCCACAGCATCCTT<br>TACCCAGCCAACTGAACATT
Lamp1	NM_010684	147	930-1076	TCTATGGCACTGCAACTGAA<br>GGCTCTGTTCTTGTTCTCCA
Lamp5	NM_029530	222	722-943	TCACGCCTACACACTCAGAA<br>TGCTGAGCCTGACACTCATA
Rab7	NM_009005	156	424-579	TGGTGCTACAGCAAAAACAA<br>GTCCAGTTTGATGGGTTCAG
Rab11fip2	NM_001033172	243	695-915	CTGGGTCCCACTTATCTTCTGA<br>GGGGTAATGAAGCAGTCACATTT
Slc26a7	NM_145947	125	55-179	CCCCACCGAGAAGACATTAAGC<br>TGAACTGCCAACATTATCCCAG
Slc4a4	NM_172830	118	10-127	CCAGGGCAGGGGGATTTTG<br>CCCCAATGTCTATGCCTGAGG
Stam	NM_011484	166	2462-2627	TGTCACCATACCAGCATACG<br>AAGGAAACCAATGCCCTTAC
Vps37a	NM_033560	202	2021-2222	CACACACACCCCTTTCTTTC<br>GACCTGTATCGCTGCCTAAA
